# Mapping the Public Health Landscape in Greece: Governance, Stakeholders, Data Systems, and Policy Frameworks

**DOI:** 10.3390/ijerph23081059

**Published:** 2026-08-14

**Authors:** Christos Triantafyllou, Anastasia Ntikoudi, Anastasia Papachristou, Vion Psiakis, Valter R. Fonseca, Joao Breda

**Affiliations:** WHO Athens Quality of Care and Patient Safety Office, 10675 Athens, Greece; ntikoudia@who.int (A.N.); papachristoua@who.int (A.P.); psiakisv@who.int (V.P.); fonsecav@who.int (V.R.F.); rodriguesdasilvabred@who.int (J.B.)

**Keywords:** public health, health services, community health, population health, health promotion, prevention programs, surveillance, health governance

## Abstract

**Highlights:**

**Public health relevance—How does this work relate to a public health issue?**

- Greece’s public health system is undergoing major post-COVID-19 transformation, while still facing persistent challenges related to regional inequalities, workforce capacity, service organization, and access to care.
- This situation analysis brings together fragmented information on Greek public health governance, institutional frameworks, stakeholders, surveillance mechanisms, and available policy tools.

**Public health significance—Why is this work of significance to public health?**

- The study provides a structured overview of the current public health landscape in Greece, supporting evidence-informed planning, policy coordination, and system-level reform.
- By mapping key actors across national, regional and local levels, the analysis highlights the importance of intersectoral collaboration for prevention, health promotion, and population health improvement.

**Public health implications—What are the key implications or messages for practitioners, policy makers and/or researchers in public health?**

- Strengthening public health in Greece requires clearer governance structures, better coordination among stakeholders, and more systematic use of high-quality, interoperable data.
- Future public health strategies should prioritize prevention, health equity, workforce development, digital transformation and locally adapted interventions for vulnerable populations.

**Abstract:**

Background: Public health in Greece has undergone substantial changes since the COVID-19 pandemic, while challenges related to governance, workforce distribution, regional inequalities and service organization remain. This study aimed to map the current public health landscape in Greece by examining its institutional frameworks, stakeholder roles, policy implementation and public health data systems. Methods: A structured situation analysis combining a review of peer-reviewed and gray literature, policy analysis, and stakeholder mapping was conducted. PubMed, EMBASE, and CINAHL were searched for English- and Greek-language publications issued between 2005 and 2026, supplemented by reports, legislation, and policy documents from the Greek Ministry of Health, the World Health Organization, the Organisation for Economic Co-operation and Development, the European Commission, and other relevant institutions. Results: Public health responsibilities were distributed across multiple national, regional, and local institutions, creating challenges concerning coordination and accountability. Regional and socioeconomic inequalities continued to affect access to services, while workforce shortages and skill-mix imbalances constrained public health capacity. Public health information was dispersed across different institutions and data systems, with limitations concerning standardization, accessibility, and interoperability. Recent legislation, prevention programs, and digital-health initiatives indicated increased policy attention to prevention and population health, although publicly available evidence regarding their implementation and outcomes remained limited. Conclusions: Strengthening public health in Greece requires clearer institutional responsibilities, improved coordination across governance levels, sustainable workforce planning and interoperable data systems that support routine monitoring of program coverage, equity, and population-level outcomes.

## 1. Introduction

Public health encompasses organized actions aimed at preventing disease, promoting health, protecting populations, and reducing avoidable inequalities. Its effective operation depends on coherent governance, intersectoral coordination, an appropriately skilled workforce, epidemiological surveillance, and the systematic use of reliable data [1,2,3,4,5,6]. These functions are particularly important in health systems facing demographic aging, chronic disease, emerging health threats, and unequal distribution of services.

In Greece, public health has been shaped by prolonged economic pressures, population aging, workforce shortages, regional disparities, and socioeconomic inequalities [7]. The COVID-19 pandemic further highlighted the importance of preparedness, surveillance, health communication, digital infrastructure, and coordination across national, regional, and local levels [8,9]. In response, recent reforms have increased policy attention to prevention, health promotion, screening, and digital transformation. Nevertheless, public health responsibilities remain distributed across multiple institutions, while relevant information is dispersed across legislation, institutional reports, academic studies, and separate data systems.

Existing studies and reports have generally examined individual components of the Greek public health and healthcare system, including access, primary care, workforce capacity, regional inequalities, and specific prevention initiatives. However, an integrated analysis of current governance arrangements, stakeholder responsibilities, policy frameworks, public health activities, and available data systems remains limited.

This study aimed to map the current public health landscape in Greece by examining its governance arrangements, principal stakeholders, legal and policy frameworks, public health activities, and available data systems. Specifically, it addressed the following questions: how are public health responsibilities distributed across national, regional, and local levels; which institutions contribute to policymaking, regulation, surveillance, financing, service delivery, research and advocacy; which policy frameworks and data systems support public health action; and which organizational, workforce, access, and data-related challenges are identified in the available evidence? As the study does not apply predefined indicators, benchmarks, or comparative criteria, it is presented as a situation analysis rather than a formal assessment of system performance.

## 2. Materials and Methods

### 2.1. Study Design and Analytical Framework

This study was conducted as a structured situation analysis of the public health landscape in Greece. It combined a purposive review of peer-reviewed and gray literature with policy and documentary analysis and stakeholder mapping. The study was not designed as a systematic or scoping review and did not aim to identify exhaustively all publications related to public health in Greece. Rather, it sought to integrate the most relevant and current evidence required to describe public health governance, institutional responsibilities, policy frameworks, service organization, workforce capacity, access and equity, prevention and health promotion, epidemiological surveillance, digital transformation, and public health data systems.

The analytical domains were informed by the WHO essential public health functions [2,3] and adapted to the objectives of the present situation analysis. Evidence was organized across the following domains: governance and legislation; institutional and stakeholder responsibilities; public health financing and service organization; workforce capacity; access and equity; prevention and health promotion; surveillance and preparedness; digital infrastructure; and data availability, accessibility, and interoperability.

### 2.2. Information Sources and Search Strategy

The original search was undertaken between August and September 2025 and was updated on 30 June 2026. PubMed, EMBASE, and CINAHL were searched for English- and Greek-language sources published between January 2005 and April 2026. The search combined country-related terms with terms corresponding to the predefined analytical domains. The principal search terms included “Greece”, “Greek”, “public health”, “health services”, “health governance”, “health policy”, “primary healthcare”, “health workforce”, “health inequalities”, “health promotion”, “prevention programmes”, “epidemiological surveillance”, “digital health”, “health information systems”, “stakeholders”, “data systems” and “interoperability”. Database-specific subject headings, including MeSH terms in PubMed, were combined with free-text title and abstract terms using the Boolean operators AND and OR.

Targeted searches were also conducted on the websites and document repositories of relevant Greek and international institutions. These included the Greek Ministry of Health, the National Public Health Organization, the Hellenic Statistical Authority (ELSTAT), the Ministry of Interior, the National Organization for the Provision of Health Services, the Agency for Quality Assurance in Health, e-Government Center for Social Security (IDIKA), the World Health Organization, the European Observatory on Health Systems and Policies, the Organisation for Economic Co-operation and Development and the European Commission. The gray-literature search included legislation, national strategies, policy documents, institutional and administrative reports, surveillance reports, official statistical information, and relevant institutional webpages. Reference lists of key academic and institutional sources were also examined to identify additional material.

### 2.3. Eligibility Criteria and Source Selection

Sources were considered eligible when they addressed at least one system-level aspect of public health in Greece. Eligible topics included public health governance and legislation; institutional and stakeholder responsibilities; service organization and financing; public health and primary healthcare workforce capacity; prevention and health-promotion policies; epidemiological surveillance and preparedness; regional, geographical, or socioeconomic inequalities; digital-health infrastructure; health information systems; data governance; and intersectoral collaboration.

Peer-reviewed publications, legislation, strategic plans, policy documents, official reports, and other relevant institutional sources were eligible for inclusion. Sources focusing exclusively on clinical management or disease-specific treatment without relevance to public health organization, governance or policy were excluded. Documents that had been superseded by more recent legislation or policy frameworks were excluded unless they were required to describe the historical development of the system. Sources outside the specified publication period, languages, or geographical scope were also excluded. Source screening and eligibility assessment were undertaken by A.N. and A.P. Potentially eligible or uncertain sources were reviewed jointly, and final inclusion or exclusion decisions were reached through discussion and consensus.

### 2.4. Data Extraction and Source Appraisal

Evidence relevant to the study objectives was identified within the included sources and integrated into the narrative analysis. Information from peer-reviewed publications, legislation, policy documents, institutional reports, and official webpages was compared to identify areas of agreement, inconsistency, and limited or missing evidence. Data extraction was undertaken by A.N. and A.P. Extracted information was checked against the original sources, and any discrepancies or uncertainties were resolved through discussion and consensus.

Given the heterogeneity of the included source types, no single formal risk-of-bias or standardized quality-assessment instrument was considered applicable. Sources were not assigned numerical quality scores. Their credibility and relevance were considered according to the authority of the issuing organization, recency, methodological transparency, relevance to the study objectives, and consistency with other available evidence. Greater interpretive weight was given to current legislation, official national and international institutional documents, and peer-reviewed publications with clearly reported methods. The absence of a formal quality-appraisal procedure was acknowledged as a methodological limitation.

### 2.5. Stakeholder Identification and Mapping

Stakeholders were identified from legislation, official organizational information, policy documents, institutional reports, and relevant academic literature. Inclusion was based on a documented role in the organization, regulation, coordination, financing, surveillance, delivery, or support of public health activities in Greece.

The identified stakeholders were described according to their level of operation and broad institutional role. These included national government and regulatory bodies, regional and local authorities, healthcare organizations, academic and research institutions, and civil-society and patient organizations. The analysis focused on their formally assigned responsibilities, their contribution to public health activities, and the main areas in which coordination with other organizations was required. The stakeholder mapping was descriptive and did not involve formal scoring, network analysis, or ranking of stakeholder influence.

### 2.6. Data Synthesis

Owing to the heterogeneity of the evidence included, statistical pooling was neither appropriate nor undertaken. The findings were synthesized using a narrative thematic approach. Evidence was compared across source types and organized under the predefined analytical domains. Where academic publications, legislation, and institutional sources provided different or potentially conflicting accounts, these differences were retained and examined rather than resolved solely through author interpretation. Areas for which current, consistent, or publicly accessible evidence was unavailable were explicitly identified as evidence gaps.

**Figure 1:**
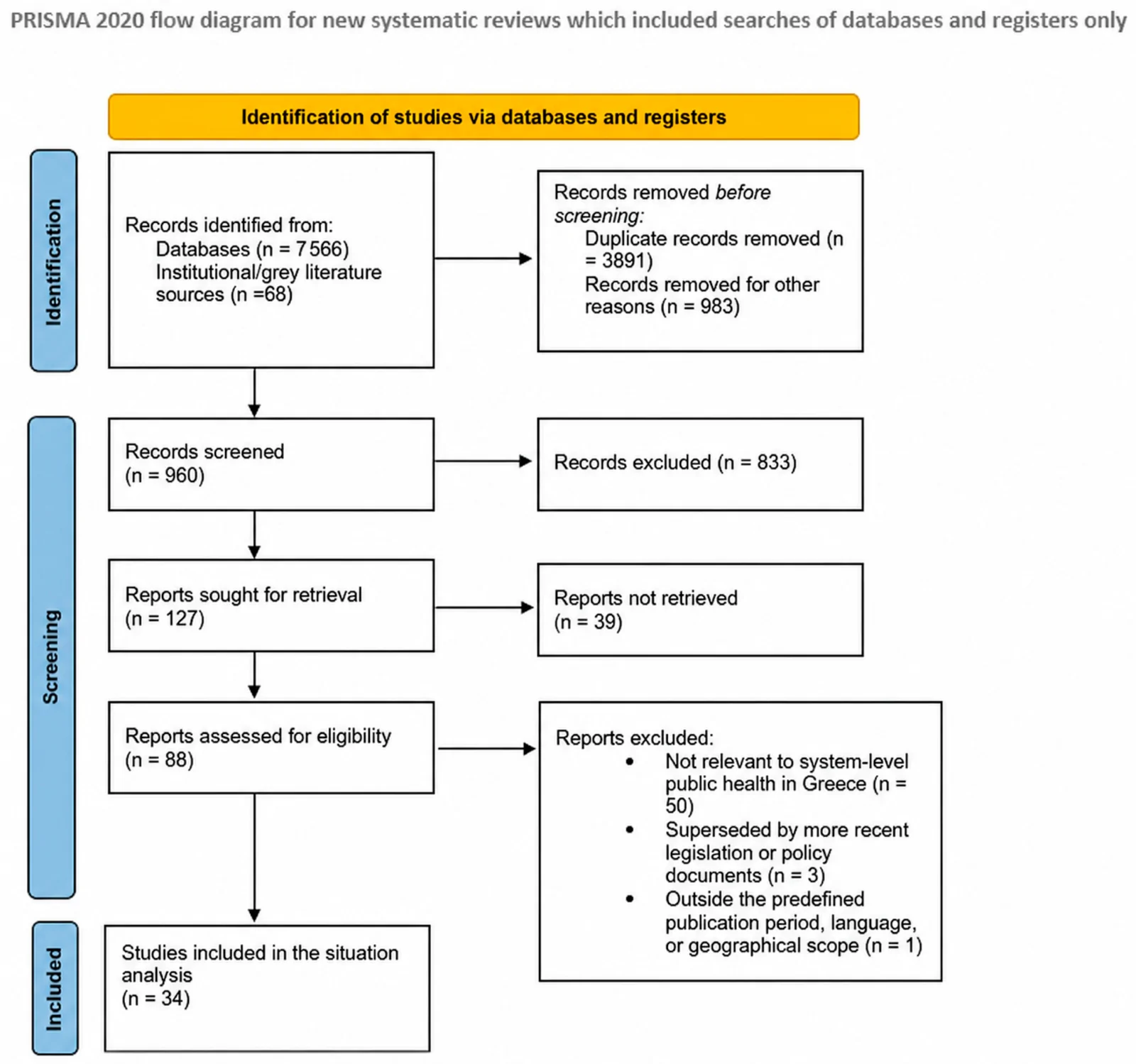
Flow of source identification and selection for the situation analysis.

The overall analytical process comprised source identification, duplicate removal, relevance assessment, detailed eligibility assessment, source appraisal, data extraction, stakeholder classification, and narrative thematic synthesis. This process is presented in Figure 1 using an adapted PRISMA-style format to improve reporting transparency.

## 3. Results

### 3.1. Current Status of Public Health Activities and Policies in Greece

#### 3.1.1. Cultural, Behavioral, and Societal Factors

Recent developments indicate increased policy attention to prevention, health promotion, screening, epidemiological preparedness, and digital health in Greece. However, the available evidence does not demonstrate a completed system-wide transition or uniform improvements across regions and population groups. The current public health landscape is therefore better characterized as one of ongoing reform, alongside persistent challenges related to resource allocation, workforce capacity, access inequalities, institutional coordination, and data fragmentation. The following subsections present the principal developments and remaining system-level challenges identified in the reviewed evidence.

#### 3.1.2. Resource Allocation

Resource allocation and funding for public health services continue to pose challenges in Greece. During the economic crisis of the early 2010s, austerity measures and reductions in public expenditure affected the capacity of the wider healthcare system [10]. Budgetary constraints have limited the resources available for public health programs and may influence the continuity and geographical consistency of preventive and health-promotion services [11]. Recent reforms have strengthened the policy focus on prevention and the more strategic use of available resources, providing a more supportive foundation for the expansion of public health programs and more balanced service provision across regions and population groups. Continued monitoring of expenditure, program coverage, and regional resource allocation is therefore required.

#### 3.1.3. Workforce Development

The healthcare system in Greece has faced challenges related to capacity, staffing, and the availability of resources [7]. Major challenges are: (a) the number of healthcare workers, especially when it comes to nurses and the ratio of generalists to specialist physicians, and (b) skills that are necessary in the modern context of the provision of public health services, from planning to delivery [7]. Recent initiatives have placed greater emphasis on workforce planning, professional training, primary healthcare teams, health promotion, and disease prevention [11]. These developments indicate increasing recognition of the competencies required for public health delivery, although workforce shortages and skill-mix imbalances remain important challenges.

#### 3.1.4. Regional Disparities

The distribution of public health services in Greece is quite uneven, with urban areas often having better access to specialized care than rural regions [12]. Promising initiatives regarding decentralization of public health services have been introduced in order to improve access to healthcare services in distant and island areas [12]. Community-based interventions, mobile health services, and telemedicine programs have been introduced as mechanisms for improving access in rural, remote, and island areas, although their coverage and effect on regional inequalities require continued evaluation [13].

#### 3.1.5. Socioeconomic Inequalities

Many differences are observed in parts of the population regarding socioeconomic factors. Income inequality is above the EU average, and there are marked differences in terms of access to services or access to information [14]. The Greek authorities work on reducing socioeconomic disparities by providing healthcare services to all citizens regardless of their financial or social insurance status. Recent reforms and outreach initiatives have increased policy attention to the needs of vulnerable populations, including access to health information and preventive services. Nevertheless, socioeconomic inequalities continue to affect effective access to healthcare and public health interventions.

#### 3.1.6. Demographic Factors

Greece has an aging population, leading to an increased demand for healthcare services, long-term care, and management of chronic conditions. Projections indicate that by 2050, more than a third of the Greek population will be older than 65 years, with nearly 13% older than 80 years [15]. Addressing the specific health needs of the elderly presents challenges for the healthcare system [7]. The public sector cooperates with municipalities and other civil society organizations in order to deliver home-based care services, supporting in this way healthy aging and chronic disease management [11].

#### 3.1.7. Refugee and Migrant Health

The country has faced various challenges in addressing the health needs of refugees and migrants. Issues include access to healthcare services, communicable disease control, and the integration of diverse populations into the healthcare system [16]. During the past decade, the public health response has increasingly involved collaboration with non-governmental and international organizations providing culturally appropriate healthcare, vaccination, and disease-prevention services [16].

Addressing these challenges requires a comprehensive and coordinated approach involving government agencies, healthcare providers, communities, and international collaborations. Continued investment in public health infrastructure, workforce development, and evidence-based policies is crucial to overcoming these barriers.

### 3.2. Stakeholders

#### 3.2.1. Public Health Stakeholders in Greece

Public health governance in Greece involves institutions operating at national, regional, and local levels. These include the Ministry of Health and its supervised agencies, the Health Regions, regional and municipal authorities, healthcare providers, academic and research institutions, professional organizations, patient associations, and civil-society organizations. Their responsibilities include policymaking, regulation, epidemiological surveillance, health-service administration, quality assurance, data management, research, health promotion, and advocacy. Table 1 summarizes the principal stakeholders, their level of operation, main responsibilities, and institutional relationships.

#### 3.2.2. Ministry of Health

The Ministry of Health is the principal national authority responsible for the formulation, implementation, and supervision of health and public health policy in Greece. Through the General Secretariat for Public Health and the General Directorate of Public Health and Quality of Life, it coordinates activities related to disease prevention, health promotion, communicable and non-communicable disease control, environmental health, public health inspections, addictions, and emergency preparedness [17]. The Ministry also oversees the wider organization of healthcare services, regulates professional and institutional standards, and supervises national agencies and Health Regions involved in public health and healthcare delivery. Because many determinants of health extend beyond the healthcare sector, the Ministry collaborates with other ministries, regional and municipal authorities, public organizations, healthcare providers, and international institutions. This coordinating role is particularly important for ensuring alignment between national policy priorities and their implementation across different levels of governance.

#### 3.2.3. Regional Level-DYPE

The regional organization of health and public health services in Greece has developed through successive legislative reforms. Laws 2503/1997 and 2519/1997 provided an early framework for the organization of regional administration and public health services, including regional responsibilities for prevention, health promotion, and the protection of population health [18,19]. Greece is currently divided into seven Health Regions, which operate as public-law entities under the supervision of the Ministry of Health and are responsible for the planning, coordination, supervision, and monitoring of healthcare organizations within their respective geographical jurisdictions [20]. The current composition and geographical jurisdiction of the seven Health Regions were specified through the replacement of Article 1 of Law 3329/2005 by Article 27 of Law 4771/2021 [20]. Health Regions constitute sector-specific health-administration bodies and should be distinguished from the country’s administrative regions, which form part of the local-government structure and exercise separate public health responsibilities.

#### 3.2.4. First and Second Levels of Local Government

Public health responsibilities at the subnational level are exercised through the two levels of local government: municipalities at the first level and the 13 administrative regions at the second level. The former prefectural administrations no longer constitute an independent level of government; their functions were transferred mainly to the regions and municipalities, while regional units now operate as territorial subdivisions of the administrative regions. Law 3370/2005 provided an earlier framework for the organization and coordination of public health services and their relationships with central and regional authorities [21]. The current local-government structure is governed by the Local Government Code introduced by Law 5314/2026 [22], building on the administrative reforms established through Law 3852/2010 [23]. Regional and municipal authorities exercise public health-related responsibilities according to their statutory competence, including environmental health, sanitary controls, prevention and health promotion, community services, and locally implemented public health activities. These responsibilities require coordination with the Ministry of Health, the Health Regions, the National Public Health Organization, and other competent bodies.

#### 3.2.5. Regional and Local Public Health Responsibilities

Greece’s subnational administrative structure comprises 13 administrative regions and municipalities, while regional units operate as territorial subdivisions of the regions. Following the administrative reform introduced by Law 3852/2010, the former prefectural administrations ceased to constitute a separate level of local government, and their institutions and responsibilities were transferred principally to the regions and municipalities [24]. Competent regional and municipal services exercise public health-related responsibilities that include the enforcement of health regulations, sanitary inspections, monitoring of water and food safety, and the licensing or supervision of establishments of public health relevance. These functions are undertaken within the statutory responsibilities of each authority and in coordination with the competent national and health-sector institutions.

Public health services of the administrative regions and municipalities contribute, within their statutory responsibilities, to vaccination activities, communicable-disease notification and surveillance, epidemiological investigation, health education, disease prevention, and health-promotion measures. These functions are implemented in coordination with the Ministry of Health, the National Public Health Organization, the Health Regions and healthcare providers [21,25].

The oversight of both public and private health services, including clinics, laboratories, pharmacies, dentists, physiotherapists, and various health-related professions such as psychologists, nurses, dental technicians, beauticians, opticians, hairdressers, and slimming centers, falls within the responsibilities of the competent regional health and public health authorities. These authorities hold the legal and institutional authority to regulate and register these professions, granting professional licenses, licenses for the establishment and operation of professional units, and certificates of good functioning, among other responsibilities [25].

The Greek healthcare system consists of primary healthcare centers and secondary and tertiary hospitals, which provide both prevention and care services. The delivery of the first-line public health services depends mainly on specialized healthcare professionals (community nurses, general practitioners, and health visitors) who facilitate public health promotion activities, perform screenings in the general population and vaccination campaigns, and deliver community health interventions [25].

### 3.3. Other Public Health Stakeholders and Supporting Agencies

At the national level, the National Organization for the Provision of Health Services (EOPYY) purchases, contracts, and reimburses publicly funded healthcare services; the Agency for Quality Assurance in Health (ODIPY S.A.) supports quality assurance and patient safety; and the National Evaluation Center of Quality and Technology in Health (EKAPTY) evaluates and certifies quality-management systems and medical devices in the health sector (Table 1).

Non-governmental organizations, in conjunction with patient advocacy groups, play a crucial role as essential components of the promotion of public health. The Patients’ Alliance, the Hellenic Cancer Federation (ELLOK) and the Forum for Public Health are entities whose purpose is to raise awareness and deliver patient support services while advocating patients’ rights.

Academic institutions and research organizations are important contributors to public health initiatives through not only their curriculum regarding public health awareness and education but also through their research work. These institutions provide scientifically substantiated evidence for policy-making purposes and contribute significantly to shaping future public health specialists.

The stakeholders described above contribute to public health policy, service delivery, research, quality improvement, advocacy, and community support. Effective implementation requires coordination among governmental bodies, healthcare organizations, academic institutions, professional organizations, and civil-society actors, with clearly defined responsibilities and communication mechanisms across national, regional, and local levels.

### 3.4. Frameworks

#### Legal and Regulatory Framework

A series of legislative efforts for the regulation of the field of public health has taken place from 2000 onwards. The main text currently regulating the field of public health in Greece (Law 4675/2020) was voted and implemented in an effort to highlight the role of public health, reduce overlap between the different bodies and institutions, and align the country with the international setting of public health [25]. The law defines (1) terms related to public health (e.g., specifies public health policies) and (2) actions that can be taken by the state in order to compile and implement those policies. Such actions include (a) the design, assessment, and socio-economic evaluation of healthcare programs and interventions, (b) the management and control of social, behavioral, and environmental risk factors for health, as well as those arising from climate change and the migration of large segments of the population. The law specifically links public health actions with primary health care, at the community level [23].

The National Action Plan for Public Health 2021–2025 [26,27] introduced priorities related to prevention programs, screening interventions, digital health transformation, and intersectoral public health governance. Additional national initiatives, including quality improvement and digital health strategies, further support the modernization and coordination of public health services in Greece.

Recent national prevention initiatives, including the ‘Spyros Doxiadis’ program, have further strengthened the implementation of primary, secondary, and tertiary prevention policies in Greece through population-based screening, health promotion, and early intervention strategies [28].

Furthermore, public health reforms in Greece have also emphasized digital transformation through the expansion of electronic health records, telehealth, and teleconsultation services, as well as digital epidemiological surveillance systems aimed at improving healthcare coordination and evidence-informed decision-making [29].

### 3.5. Available Public Health Data Systems and Metrics

Public health information in Greece is generated through statistical, epidemiological, clinical, and administrative data systems managed by different institutions. The Hellenic Statistical Authority (ELSTAT) produces population-level information through the Health Interview Survey and other official health-statistics collections. The 2019 Health Interview Survey provides published national results, while ELSTAT also permits access to anonymized public-use and scientific-use microdata, including National Health Survey and mortality data, through established registration, application, and approval procedures [30,31]. These sources are updated according to the schedule of each survey or statistical series and primarily support population-level analysis and national and international comparisons rather than real-time surveillance.

The National Public Health Organization (EODY) operates the mandatory notification system and other disease-specific epidemiological-surveillance mechanisms. Notifications submitted by healthcare facilities are received, recorded, and analyzed by the competent EODY directorates and are used to support public health interventions [32]. The system is updated as notifications are submitted, according to the reporting requirements and deadlines applicable to each notifiable disease. Access to identifiable case-level information is restricted, while aggregate findings are disseminated through epidemiological reports. The completeness and timeliness of the data depend partly on reporting by healthcare facilities and professionals.

IDIKA S.A. operates important components of the national digital-health infrastructure, including electronic prescribing and the National Electronic Health Record. Electronic-prescribing data are generated and updated through the routine issue and execution of prescriptions [33]. The National Electronic Health Record is designed to integrate information from electronic prescribing, primary healthcare, hospitals, diagnostic centers, patient registries, and EOPYY [34]. Its national implementation through the MyHealth platform version 2.12.32 commenced in 2025, providing citizens and authorized healthcare professionals with access to integrated health information [35].

EOPYY generates administrative and reimbursement data through its purchasing and contracting functions. Its Health Insurance Record contains information on hospitalizations, healthcare services, medical materials, diseases, and diagnoses, derived from information submitted by healthcare providers and insured persons for the reimbursement of healthcare benefits [36]. These records are updated through routine service and reimbursement submissions and cover a substantial proportion of the insured population. Individual insured persons may access their own records, but the source does not indicate general public access to record-level data for research or population-health monitoring. Because these data are collected primarily for administrative and reimbursement purposes, their secondary use for epidemiological analysis requires appropriate validation and standardized coding.

Overall, Greece has several data systems with broad population and service coverage, but these systems differ in purpose, update frequency, accessibility, and data structure. The National eHealth Interoperability Framework and the National Electronic Health Record represent important developments towards more standardized data exchange and integration [34,35,37]. Nevertheless, the reviewed public sources do not provide a single cross-system assessment of data completeness, coding consistency, linkage quality, and interoperability. These dimensions should therefore be considered when statistical, surveillance, clinical, and reimbursement data are reused for program evaluation, research, and public health decision-making.

## 4. Discussion

The findings indicate that Greece has increased policy attention to prevention, digital health and public health coordination, but the available evidence does not yet demonstrate uniform implementation or measurable improvement across all regions and population groups. This interpretation is consistent with the latest Greek Country Health Profile, which identifies continuing pressures related to service delivery, workforce capacity, infrastructure, preparedness, digital transformation, and long-term system sustainability [38]. Similar challenges are reported in other Southern European health systems, although their governance arrangements differ. Spain operates a highly decentralized system in which regional authorities hold substantial responsibility for service organization and delivery, supported by national coordination through the Interterritorial Council [39]. Portugal has pursued stronger central management while simultaneously expanding primary and integrated care [40]. These comparisons suggest that the involvement of multiple administrative levels does not necessarily result in fragmentation; the critical factors are the clarity of institutional responsibilities, effective coordination mechanisms, accountability, and the capacity to share and use information across organizations.

Management of chronic diseases is another factor that plays an important role in the Greek public health governance [41]. The following years require targeted health policy planning focused on the prevention and management of chronic diseases, long-term care, and community-based services, as the population changes over the years have led to increased requirements for improved public health initiatives [41].

The digital transformation of the Greek healthcare system creates not only new opportunities but major challenges for public health services [42]. The implementation of digital tools such as electronic health records, telehealth/teleconsultations, and digital epidemiological surveillance systems provides additional resources to healthcare governance in order to improve the operational performance and data-oriented decision-making [42].

The Greek public health sector has undergone important reforms in recent years; however, challenges related to institutional coordination, workforce capacity, and consistency in service delivery remain [42]. Against this background, the HEALTH-IQ project represents one of the current national efforts to strengthen quality of care and patient safety. Coordinated by the WHO Athens Quality of Care and Patient Safety Office in collaboration with the Greek Ministry of Health, the project aims to support the development of a more standardized quality framework across health services [43,44]. At the time of this analysis, publicly available information focused mainly on the project’s objectives, governance arrangements, and planned activities, while independent evidence regarding its outcomes and system-level impact remained limited. HEALTH-IQ should therefore be understood as an ongoing policy and implementation initiative rather than as evidence of demonstrated improvement.

Standardization of data poses a major obstacle in the field of public health. The evaluation of public health policies and evidence-based decision-making depends mainly on the availability of high-quality data, which must be timely and comparable. The implementation of public health initiatives requires ongoing interdisciplinary teamwork among healthcare professionals and epidemiology experts [45,46].

Public health services need to be defined by cultural competence and participatory methods in order to design and implement programs which meet the population’s needs, including marginalized groups [47]. Public health strategies need to be adaptive and evidence-based to be able to handle emerging health threats, including pandemics and climate-related emergencies [47]. The public healthcare workforce requires specialized professionals in public health, epidemiology, and health promotion in order to maintain high-quality health services in the Greek population [48].

This study has several strengths and limitations. Its principal strength is the integration of peer-reviewed literature, legislation, policy documents, institutional reports, and stakeholder information within a common analytical framework, providing a broad overview of public health governance and data systems in Greece. However, the purposive rather than exhaustive selection of sources may have introduced selection bias, particularly where relevant documents were not publicly accessible or were not identified through the selected databases and institutional websites. The substantial contribution of gray literature was necessary because many governance and policy developments are documented primarily in legislation and institutional publications; nevertheless, these sources vary in methodological transparency and may reflect the priorities of the organizations that produced them. No formal quality-assessment tool or numerical weighting system was applied because of the heterogeneity of the included evidence, which limits the ability to compare the methodological strength of individual sources. In addition, the rapidly changing legislative, organizational, and digital-health environment means that some findings may become outdated as reforms progress. The results should therefore be interpreted as a structured account of the evidence available during the review period rather than as a definitive evaluation of current system performance.

## 5. Conclusions

Based on the evidence reviewed, Greece appears to be moving towards a more prevention-oriented, digitally supported, and coordinated approach to public health, while important challenges remain in governance, workforce capacity, regional equity and data interoperability. Recent reforms and initiatives provide opportunities for further system strengthening, but their implementation, coverage and outcomes require continued and independent evaluation. Future progress will depend on sustainable resource allocation, clearer institutional responsibilities, effective coordination across governance levels and the routine use of comparable public health data. The findings should therefore be interpreted as a current mapping of policy and system developments rather than as evidence that a comprehensive and equitable public health system has already been achieved.

## Figures and Tables

**Table 1:** Principal stakeholders in the Greek public health system.

Stakeholder	Governance Level	Main Role and Authority	Main Institutional Relationships
Ministry of Health	National	Develops and supervises health and public health policy, legislation, regulation and strategic planning	Supervises national agencies and Health Regions and coordinates with other ministries and international organizations
National Public Health Organization	National	Epidemiological surveillance, disease prevention, risk assessment, preparedness and outbreak response	Cooperates with the Ministry of Health, Health Regions, laboratories and healthcare providers
Health Regions (YPE/DYPE)	Regional health administration	Plan, coordinate, supervise and monitor healthcare organizations within their jurisdictions	Operate under the Ministry of Health and coordinate hospitals, health centers and primary healthcare services
Administrative regions and municipalities	Regional and local government	Environmental health, sanitary controls, health promotion, prevention and community services	Cooperate with the Ministry of Health, Health Regions, national agencies and local providers
EOPYY	National	Purchases, contracts and reimburses publicly funded healthcare services	Cooperates with the Ministry, public and private providers and national digital systems
ODIPY S.A.	National	Supports quality assurance, quality improvement and patient safety	Cooperates with the Ministry, Health Regions and healthcare organizations
EKAPTY	National	Supports quality evaluation and certification activities in the health sector	Cooperates with health authorities, providers and relevant regulatory bodies
Academic and research institutions	National and regional	Research, evidence generation, education and workforce development	Cooperate with public authorities, healthcare organizations and research networks
Patient and civil-society organizations	National and local	Advocacy, patient representation, public information and community support	Cooperate with policymakers, providers, researchers and local communities

## Data Availability

No new data were created or analyzed in this study. Data sharing is not applicable to this article.

## Abbreviations

The following abbreviations are used in this manuscript:

DYPE	Regional Health Authorities/Health Regions
EKAPTY	National Evaluation Center of Quality and Technology in Health
ELLOK	Hellenic Cancer Federation
EOPYY	National Organization for the Provision of Health Services
EU	European Union
HEALTH-IQ	Health Information Quality Project
NGOs	Non-governmental organizations
OECD	Organisation for Economic Co-operation and Development
ODIPY S.A.	Agency for Quality Assurance in Health S.A.
OTA	Local Government Authorities
WHO	World Health Organization
YPE	Regional Health Authorities/Health Regions
